# Multimodal Learning with Privileged Report Supervision for Generalizable Tuberculosis Detection on Chest Radiographs

**DOI:** 10.1007/s10916-026-02368-3

**Published:** 2026-04-09

**Authors:** Sivaramakrishnan Rajaraman, Niccolo Marini, Zhaohui Liang, Zhiyun Xue, Sameer Antani

**Affiliations:** Division of Intramural Research, National Library of Medicine, National Institutes of Health, Bethesda, MD 20894 USA

**Keywords:** Tuberculosis, Deep learning, Multimodal, Classification, Privileged supervision, Generalization, Triaging

## Abstract

Multimodal learning using images and associated clinical text offers richer semantic supervision for medical AI. However, models trained with synthetic reports risk hallucination, and conventional multimodal tuberculosis (TB) systems are impractical because they require text at inference. In realworld screening workflows, particularly in low-resource settings or during triage, radiology reports are often unavailable or delayed. Computer-aided detection systems for chest X-rays (CXRs) are considered a potential solution. In this context, this study proposes a method that uses clinically grounded text as privileged information during training to improve a binary CXR classifier, while enabling image-only TB prediction at deployment. Frontal CXRs from Shenzhen (internal train/validation/test), Montgomery County, TBX11K, and NIAID TB Portals (external tests) were lung-cropped using a YOLOv8s detector and resized to 224 × 224. For Shenzhen, de-identified metadata and brief clinical notes were converted into structured reports encoding population type, TB status, laterality, lobar involvement, and adjunct findings; a parallel model used raw notes. A VGG-11 vision encoder and frozen CXR BERT text encoder were co-trained in a shared 256-dimensional space using image classification, cosine similarity, and supervised contrastive alignment losses. At inference, the text branch was removed, yielding an image only classifier regularized through multimodal supervision. Multimodal training with report supervision consistently improved image-only predictions, with structured report outperforming raw notes. Across internal and external cohorts, performance gains were reflected in higher balanced accuracy, Matthews correlation coefficient, and area under the curve. UMAP embeddings showed clearer class separation, and Grad CAM maps demonstrated improved localization of TB-relevant lesions.

**Supplementary Information:**

The online version contains supplementary material available at 10.1007/s10916-026-02368-3.

## Introduction

Multimodal learning has become increasingly important in medical artificial intelligence (AI) because clinicians rarely rely on images in isolation. Radiographs, CT scans, and other imaging studies are routinely interpreted alongside free‑text reports, structured clinical variables, laboratory findings, and broader patient context. Multimodal models that learn jointly from these complementary sources can capture relationships that are difficult to infer from images alone, leading to more robust and clinically meaningful representations and improved downstream performance. [1, 2] This has been demonstrated in chest X-ray (CXR) imaging, where pairing CXRs with their accompanying reports has enabled retrieval, automated report generation, and prompt‑based classification, and where recent foundation‑model work highlights the promise of large‑scale multimodal pretraining. [3, 4]

Tuberculosis (TB), however, poses a distinctive practical challenge for multimodal deployment. TB remains a major global health threat, with substantial morbidity and mortality despite effective therapies. [5] CXR imaging plays a central role in screening, diagnosis, and treatment monitoring, including in settings where microbiological confirmation is delayed or unavailable. [6, 7] Deep learning (DL) systems have shown strong performance for CXR classification and TB triage, motivating real-world CAD deployment where radiology expertise is limited. [8–10] Yet TB datasets are typically smaller and more heterogeneous than general CXR corpora, reflecting limited sites, devices, and populations; this heterogeneity makes cross-dataset generalization a persistent challenge. [11]

Critically, most TB-focused public datasets do not provide paired radiology reports in a form that supports multimodal learning, and even when notes exist, they are often used only to derive labels rather than to support robust image-text modeling. [12, 13] Beyond dataset availability, real-world screening workflows, especially in low-resource settings or during triage, often require CAD systems to operate on the image alone, because radiology reports may be missing, delayed, or inconsistent. [2, 4] This makes many conventional multimodal TB systems impractical at deployment time, despite their potential benefits during training.

One might attempt to fill the missing-text gap by generating synthetic reports with large language models (LLMs), but safety concerns arise because LLM-based report generation can hallucinate plausible but incorrect findings, particularly under incomplete context or distribution shift. [14, 15] Prior evaluations have shown that automated report generation can diverge from expert judgment by omitting key abnormalities or introducing unsupported statements. [4, 15] Such hallucinations are unacceptable for TB screening because they can mislead downstream decisions and compromise clinical trust.

These constraints motivate an alternative framing: using non-image information as privileged supervision during training only to improve image-only inference. Vapnik’s Learning Using Privileged Information (LUPI) framework formalizes this idea by allowing a learner to train on standard inputs 𝑥 (images) alongside privileged inputs 𝑥* (clinical text) that are available only during training, with theory showing that privileged information can alter the optimal solution and improve generalization. [16] LUPI-style concepts have been applied in medical imaging using privileged supervision, such as uncertainty signals or semi-paired image-text data. [17, 18] However, to our knowledge, LUPI-style training that uses deterministic, structured clinical text derived from de-identified metadata and brief clinical notes as privileged supervision has not been systematically studied for TB detection on CXRs nor evaluated for generalization across external TB cohorts.

In this work, we propose an image text alignment-regularized strategy under the LUPI paradigm to strengthen deployable image-only TB prediction. During training, deterministic PII-safe structured reports derived from de-identified metadata and brief clinical notes provide privileged supervision that steers the vision backbone toward clinically grounded representations; at inference, the text branch is discarded, so deployment remains strictly image-only. We compare two privileged-text regimes raw clinical notes versus structured reports to isolate the role of text quality in multimodal regularization. We train on Shenzhen CXRs using internal train/validation/test splits and assess generalization on external Montgomery County, TBX11K, and NIAID TB Portals cohorts. We further examine representational and localization shifts induced by privileged text via UMAP embeddings and Grad-CAM saliency compared with available expert lesion boxes. [19, 20] This contribution offers two practical advantages: it uses deterministic, de-identified structured text rather than synthetic or potentially hallucinated reports, and it yields a deployable CXR-only model aligned with TB triage workflows where reports are often unavailable or delayed. Overall, the framework connects multimodal alignment with privileged-information training to deliver robust image-only gains for TB detection.

## Materials and Methods

### Datasets

We utilized the following publicly available CXR collections focused on pulmonary TB: the Shenzhen Hospital CXR set [9] and the Montgomery County CXR set, [9] both distributed by the U.S. National Library of Medicine (NLM), the Tuberculosis X‑ray (TBX11K) dataset [21] developed by Nankai University, and the NIAID TB Portals dataset published by the National Institute of Allergy and Infectious Diseases (NIAID). [22] Both NLM and NIAID are part of the U.S. National Institutes of Health (NIH).

Shenzhen Hospital CXR (Shenzhen CXR) dataset [9]: Collected at Shenzhen No. 3 People’s Hospital, Guangdong Medical College, China, this dataset includes 662 frontal CXRs, with 326 normal and 336 TB‑positive cases. Images were acquired using a Philips DigitalDiagnost digital radiography system and distributed as PNG files with native resolution of approximately 3,000 × 3,000 pixels. The images and metadata were de‑identified, and public release was exempted from IRB review. Each CXR image is accompanied by a text file containing the patient’s age, gender, and a brief deidentified clinical note describing the pulmonary abnormality. We compiled these attributes into a table for structured‑report generation. The original release did not include lesion masks or bounding boxes. Subsequently, Yang et al. [23] published extended annotations for the 336 TB cases, providing pixel‑level segmentations of abnormal regions. These were delineated as polygons using the Firefly web‑based labeling tool, saved as text files, and later converted into standardized JSON annotations and co‑registered binary masks aligned with the original images. We computed and stored the bounding box coordinates corresponding to these masks for further analysis.

Montgomery County CXR (MC CXR) dataset [9]: This deidentified collection was assembled in collaboration with the Department of Health and Human Services, Montgomery County, Maryland, USA, from their TB screening program. It contains 138 postero‑anterior frontal CXRs, including 80 normal and 58 TB‑positive cases. Images were acquired using a Eureka stationary computed radiography system and distributed in 12‑bit grayscale PNG format, with native resolutions of 4,020 × 4,892 or 4,892 × 4,020 pixels. Radiologist‑supervised binary lung masks are provided for both lungs, stored as separate PNG files aligned with the source images. As with the Shenzhen dataset, for each image, a paired text file with the same base name and a .txt extension stores the deidentified radiology reading, including the patient’s age, gender, and a brief description of the lung abnormality.

TBX11K CXR dataset [21]: TBX11K comprises 11,200 CXR images categorized into six classes: normal (*n* = 5,000), non‑TB but abnormal (*n* = 5,000), active TB (*n* = 924), latent TB (*n* = 212), combined active and latent TB (*n* = 54), and uncertain TB (*n* = 10). Each image was validated using diagnostic microbiology and annotated by radiologists for TB manifestations. All images were deidentified, and the dataset release was exempt from institutional review. Due to storage and computational constraints, the original radiographs were not distributed; instead, resampled 512 × 512 images were released. TB‑lesion bounding box annotations for 799 resampled TB CXRs were published in JSON and XML formats.

NIAID TB Portals CXR dataset [22]: The NIAID TB Portals Program is a multinational TB data-sharing initiative that provides an open-access, web-based repository of deidentified patient-level information, including socioeconomic, clinical, laboratory, radiological, and genomic data. The CXR collection comprises frontal-view radiographs, collected from 5,038 TB patients, with multiple images per patient reflecting longitudinal follow-up. The images are acquired under heterogeneous acquisition settings and resolutions ranging from 206 × 115 to 4453 × 3719 pixels. Each CXR is linked to structured metadata, including a unique patient identifier, age of onset, gender, treatment outcome (completed, cured, died, failure, unknown, still on treatment, lost to follow-up, or palliative care), and drug-resistance status. Drug resistance is categorized as drug-sensitive TB, mono-drug resistant TB (Mono-DR), poly-drug resistant TB (Poly-DR), multidrug-resistant TB without fluoroquinolone resistance (MDR TB, non-XDR), MDR TB with additional fluoroquinolone resistance (pre-XDR TB), or extensively drug-resistant TB (XDR TB). We randomly sampled 200 CXRs at the patient level for external validation. This evaluation provides a stringent test of how well models trained on normal/TB cohorts generalize to a clinically challenging distribution of confirmed TB cases spanning diverse resistance profiles, demographics, and acquisition conditions.

### Data Preprocessing

The CXR images were preprocessed to confine learning to the lungs and to standardize input size. Lung fields were localized using an in‑house YOLOv8s detector, [24] and were cropped to the predicted bounding boxes, thereby excluding non‑pulmonary regions and focusing on parenchymal areas where TB manifests. Cropped images were resized to 224 × 224 pixels with area interpolation, balancing diagnostic detail with computational efficiency. TB‑lesion bounding box coordinates in the Shenzhen and TBX11K datasets were correspondingly rescaled and stored.

The CXR images in the internal Shenzhen CXR dataset were split at the patient level into 70% training, 20% validation, and 10% testing. Splits were class-stratified to preserve the proportions of normal and TB cases, with rounding applied to training and validation counts and any remainder assigned to the test set. All images from a given patient were restricted to a single subset to prevent leakage, and the same assignment was applied to raw clinical notes and structured reports to maintain consistent image–text pairing. A fixed seed ensured reproducibility of the partitions.

During training, a standardized augmentation and normalization pipeline was applied. Contrast‑limited adaptive histogram equalization (CLAHE) enhanced local contrast and sharpened structures to improve generalization by reducing sensitivity to dataset‑specific lighting or contrast conditions. Images were normalized channel‑wise and converted to tensors. Validation and test transforms were deterministic, applying only resizing and normalization without CLAHE augmentation. We applied inverse‑frequency sampling during training to mitigate class imbalance. Class distributions were computed, and each image was weighted by the inverse of its class frequency. Samples were drawn with replacement to preserve dataset size while ensuring balanced exposure across classes. Validation and test loaders retained the natural distribution, allowing unbiased evaluation. Table 1 summarizes the distribution across training, validation, and test sets for internal and external data.

**Table 1:** Internal and external data distribution across train, validation, and test sets

Dataset	Train		Validation		Test		Total	
	TB	Normal	TB	Normal	TB	Normal	TB	Normal
Shenzhen CXR	235	228	67	65	34	33	336	326
MC CXR	-	-	-	-	58	80	58	80
TBX11K CXR	-	-	-	-	799	3800	799	3800
NIAID TB Portals					200	-	200	-

### Structured Report Generation from Clinical Notes

We built a deterministic, PII-safe pipeline to generate structured reports from clinical notes for every CXR in the internal Shenzhen set. The input is a complete one-row-per-image metadata table containing the filename, class label (normal or TB), numeric age in years, and a brief accompanying clinical note; no rows or fields were missing. Ages were quantized into clinically meaningful but non-identifying strata, “pediatric” (< 18 years) or “adult” (≥ 18 years). Notes were searched for predefined TB keywords, lesion locations, and adjunct pleural/parenchymal findings. For both classes (normal and TB), the structure of the report begins with the same leading clause, “This {adult/pediatric} chest radiograph,” which anchors the sentence while keeping age PII-safe. For normals, the pipeline emits a single fixed sentence: “This {adult/pediatric} chest radiograph, shows normal lungs.” The clinical note is consistent with the normal label, ensuring a uniform TB-negative representation. Representative input-to-output mappings are shown in Table 2.

**Table 2:** Examples of input fields and corresponding structured reports

Input field / variable	Structured report generated
Age = 5, Class = “normal”	“This pediatric chest radiograph, shows normal lungs.”
Age = 35, Class = “normal”	“This adult chest radiograph, shows normal lungs.”
Age = 2, Class = “TB”, note: “secondary ptb in the bilateral upper fields”	“This pediatric chest radiograph, shows secondary tuberculosis on both upper lung lobes.”
Age = 35, Class = “TB”, note: “atb”	“This adult chest radiograph, shows active tuberculosis.”
Age = 10, Class = “TB”, note: “left ptb, pleural thickening”	“This pediatric chest radiograph, shows tuberculosis on the left lung with pleural thickening.”
Age = 16, Class = “TB”, note: “secondary ptb in the left upper field”	“This pediatric chest radiograph, shows secondary tuberculosis in the left upper lobe.”
Age = 24, Class = “TB”, note: “natb”	“This adult chest radiograph, shows inactive tuberculosis.”

For TB cases, rule-based pattern matchers derive structured components from predefined lexical patterns in the note: (i) population type (adult/pediatric), (ii) TB status when explicitly stated as active or inactive, iii) TB sub-type when explicitly stated as secondary, (iv) coarse laterality and/or lobar distribution, and (iv) adjunct features such as pleurisy, pleural effusion, fibrous or hyperplastic changes, pleural adhesions/thickening, pleural change after decortication, and cavity formation, if present. Lexical variants like “tb” and “ptb” are normalized to a canonical TB token that deterministically maps to the class “tuberculosis.” If the note specifies active TB as status (e.g., “atb,” “active TB,” or “active”), inactive TB (e.g., “natb,” “inactive TB,” or “inactive”), and/or secondary TB as sub-type (e.g., “stb” or “secondary”), these information are inserted immediately after the leading clause (e.g., “This adult chest radiograph, shows inactive tuberculosis…”; “This adult chest radiograph, shows secondary tuberculosis….”). Location phrases are extracted from predefined patterns and normalized to canonical wording (e.g., “bilateral upper fields” → “on both upper lung lobes,” “in the right upper field” → “in the right upper lobe,” “left ptb” without lobar detail → “on the left lung”). Adjunct findings are deduplicated, ordered consistently, and expressed with side- or lobe-specific wording when available (e.g., “cavity formation in the right upper lobe”). The final template preserves the leading clause and comma, “This {adult/pediatric} chest radiograph, shows {status}{sub-type} tuberculosis …”, followed by an optional location fragment and then adjuncts introduced by “with,” joined by commas. If the note lacks resolvable status, sub-type, location, or adjunct details, those components are omitted, yielding a generic sentence “This {adult/pediatric} chest radiograph, shows tuberculosis.” Each report is linked to its source image by the filename stem, and one plain-text file is written per image. Because the generator is purely rule-based, rerunning it on the same inputs produces identical outputs, supporting reproducibility and straightforward debugging.

### Model Architecture

**Fig. 1:**
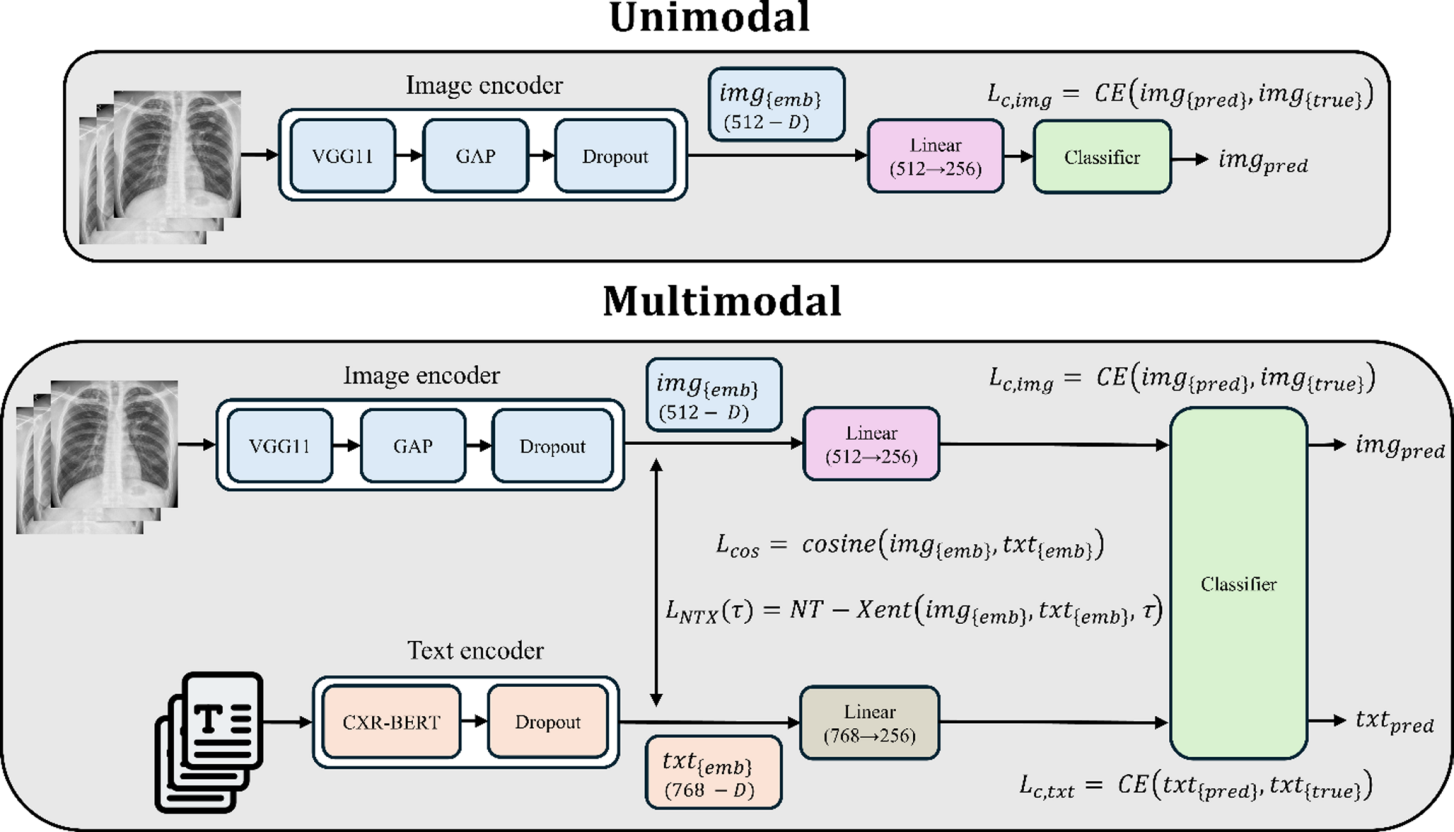
Unimodal and multimodal architecture

We trained two model families: a unimodal image-only baseline and a multimodal image–text model evaluated in two separate runs, one using raw clinical notes and the other using structured reports. This separation clarifies the role of text quality. Figure 1 summarizes both unimodal and multimodal architectures. We used the VGG-11 [25] vision backbone with batch normalization, initialized from ImageNet, and adapted to grayscale frontal CXRs by replicating the single channel to three channels. VGG-11 was selected because VGG backbones are among the most extensively validated CNNs for CXR analysis and have repeatedly shown competitive, sometimes state-of-the-art, performance. [26] Comparative studies further suggest that relatively shallow networks such as VGG can match or exceed deeper architectures on CXRs, making them strong and well-understood baselines for lung disease classification. [26] VGG variants have also served as feature extractors in downstream thoracic tasks, including COVID-19 prognosis, lung-field segmentation, and geometric preprocessing, reinforcing their suitability for representing CXR anatomy. [27, 28] However, the overall alignment framework is backbone-agnostic; any modern CNN or vision transformer could replace VGG-11 without changing the multimodal training strategy. The convolutional blocks of VGG-11 are followed by global average pooling and dropout (rate = 0.3), producing a 512-D image feature vector ${\mathbf{h}}_{i}^{\mathrm{i}\mathrm{m}\mathrm{g}}$. A linear projection maps ${\mathbf{h}}_{i}^{\mathrm{i}\mathrm{m}\mathrm{g}}\in{\mathbb{R}}^{512}$ to a 256-D embedding ${\mathbf{z}}_{i}^{\mathrm{i}\mathrm{m}\mathrm{g}}\in{\mathbb{R}}^{d}$ ($d=256$), which is passed through a classification head to yield logits ${\mathbf{o}}_{i}^{\mathrm{i}\mathrm{m}\mathrm{g}}\in{\mathbb{R}}^{2}$over “normal” and “TB.” The multimodal network incorporates a text encoder that maps clinical text into the same 256-D latent space as the image branch and shares the classification head. We use CXR-BERT, [29] a transformer pretrained on biomedical abstracts and MIMIC-III/MIMIC-CXR reports, which has demonstrated strong performance on radiology vision–language tasks. Its pooled output, followed by a 0.1-rate dropout, yields a 768-D text feature vector ${\mathbf{h}}_{i}^{txt}$. A linear layer projects ${\mathbf{h}}_{i}^{txt}\in{\mathbb{R}}^{768}$ into the shared 256-D embedding ${\mathbf{z}}_{i}^{txt}\in{\mathbb{R}}^{256}$, which is passed to the shared classification head to produce text logits ${\mathbf{o}}_{i}^{txt}\in{\mathbb{R}}^{2}$. The CXR-BERT encoder is frozen to prevent overfitting given the limited training data. We trained the multimodal model under two conditions: (i) *multimodal-raw*, where the text input is the original free-form clinical note, and (ii) *multimodal-structured*, where notes were converted into deterministic PII-safe structured reports. At inference, the text encoder is ignored in both cases, so inference uses only CXRs, with the image-head benefiting from privileged text supervision during multimodal training.

### Loss Function

Let $({\mathrm{x}}_{\mathrm{i}}^{\mathrm{i}\mathrm{m}\mathrm{g}},{\mathrm{x}}_{\mathrm{i}}^{\mathrm{t}\mathrm{x}\mathrm{t}},{\mathrm{y}}_{\mathrm{i}})$ denote the image, text, and binary label for sample $\mathrm{i}$, where $\mathrm{i}=1,\dots,\mathrm{N}$ indexes samples and ${\mathrm{y}}_{\mathrm{i}}$ is encoded as a one-hot label vector ${\mathbf{y}}_{\mathrm{i}}\in\{\mathrm{0,1}{\}}^{2}$over the two classes (normal, TB). The image encoder maps ${\mathrm{x}}_{\mathrm{i}}^{\mathrm{i}\mathrm{m}\mathrm{g}}$ to an embedding ${\mathbf{z}}_{\mathrm{i}}^{\mathrm{i}\mathrm{m}\mathrm{g}}$, and the text encoder maps ${\mathrm{x}}_{\mathrm{i}}^{\mathrm{t}\mathrm{x}\mathrm{t}}$ to an embedding ${\mathbf{z}}_{\mathrm{i}}^{\mathrm{t}\mathrm{x}\mathrm{t}}$. Both embeddings were passed through the same shared classifier $\mathrm{g}(\cdot)$ to produce unnormalized scores (logits) ${\mathbf{o}}_{\mathrm{i}}^{\mathrm{i}\mathrm{m}\mathrm{g}}=\mathrm{g}\left({\mathbf{z}}_{\mathrm{i}}^{\mathrm{i}\mathrm{m}\mathrm{g}}\right)$and ${\mathbf{o}}_{\mathrm{i}}^{\mathrm{t}\mathrm{x}\mathrm{t}}=\mathrm{g}\left({\mathbf{z}}_{\mathrm{i}}^{\mathrm{t}\mathrm{x}\mathrm{t}}\right)$, each in ${\mathbb{R}}^{2}$. These logits are raw confidence scores for the two classes before conversion to probabilities. For the image classification loss, we first converted the image logits into class probabilities using the Softmax function. For each sample $\mathrm{I}$ and class $\mathrm{k}\in\left\{\mathrm{1,2}\right\}$,

1 $${\mathrm{p}}_{\mathrm{i}\mathrm{k}}^{\mathrm{i}\mathrm{m}\mathrm{g}}=\mathrm{S}\mathrm{o}\mathrm{f}\mathrm{t}\mathrm{m}\mathrm{a}\mathrm{x}({\mathbf{o}}_{\mathrm{i}}^{\mathrm{i}\mathrm{m}\mathrm{g}}{)}_{\mathrm{k}}=\frac{\mathrm{e}\mathrm{x}\mathrm{p}\left({\mathrm{o}}_{\mathrm{i}\mathrm{k}}^{\mathrm{i}\mathrm{m}\mathrm{g}}\right)}{\sum_{\mathrm{j}=1}^{2}\mathrm{e}\mathrm{x}\mathrm{p}\left({\mathrm{o}}_{\mathrm{i}\mathrm{j}}^{\mathrm{i}\mathrm{m}\mathrm{g}}\right)}$$

Here, ${\mathrm{o}}_{\mathrm{i}\mathrm{k}}^{\mathrm{i}\mathrm{m}\mathrm{g}}$ in Eq. (1) is the ${k}^{th}$ component of ${\mathbf{o}}_{\mathrm{i}}^{\mathrm{i}\mathrm{m}\mathrm{g}}$. The image classification loss ${\mathrm{L}}_{\mathrm{c},\mathrm{i}\mathrm{m}\mathrm{g}}$ is given by the average cross-entropy between the true labels and the predicted probabilities, as shown below:

2 $${\mathrm{L}}_{\mathrm{c},\mathrm{i}\mathrm{m}\mathrm{g}}=-\frac{1}{\mathrm{N}}\sum_{\mathrm{i}=1}^{\mathrm{N}}\sum_{\mathrm{k}=1}^{2}{\mathrm{y}}_{\mathrm{i}\mathrm{k}}{\hspace{0.17em}}\mathrm{l}\mathrm{o}\mathrm{g}{\mathrm{p}}_{\mathrm{i}\mathrm{k}}^{\mathrm{img}}$$

Here, ${\mathrm{y}}_{\mathrm{i}\mathrm{k}}=1$ if sample $\mathrm{i}$ belongs to class $\mathrm{k}$ (normal or TB) and ${\mathrm{y}}_{\mathrm{i}\mathrm{k}}=0$ otherwise. This loss penalizes the model whenever the predicted probability ${\mathrm{p}}_{\mathrm{i}\mathrm{k}}^{\mathrm{i}\mathrm{m}\mathrm{g}}$ assigned to the true class is low, encouraging the image branch to assign high probability to the correct diagnosis. The text classification loss is defined in the same way but applied to the text logits and corresponding probabilities. We computed text probabilities and the text cross-entropy loss is computed as,

3 $${\mathrm{p}}_{\mathrm{i}\mathrm{k}}^{\mathrm{t}\mathrm{x}\mathrm{t}}=\mathrm{s}\mathrm{o}\mathrm{f}\mathrm{t}\mathrm{m}\mathrm{a}\mathrm{x}({\mathbf{o}}_{\mathrm{i}}^{\mathrm{t}\mathrm{x}\mathrm{t}}{)}_{\mathrm{k}}=\frac{\mathrm{e}\mathrm{x}\mathrm{p}\left({\mathrm{o}}_{\mathrm{i}\mathrm{k}}^{\mathrm{t}\mathrm{x}\mathrm{t}}\right)}{\sum_{\mathrm{j}=1}^{2}\mathrm{e}\mathrm{x}\mathrm{p}\left({\mathrm{o}}_{\mathrm{i}\mathrm{j}}^{\mathrm{t}\mathrm{x}\mathrm{t}}\right)}$$

4 $${\mathrm{L}}_{\mathrm{c},\mathrm{t}\mathrm{x}\mathrm{t}}=-\frac{1}{\mathrm{N}}\sum_{\mathrm{i}=1}^{\mathrm{N}}\sum_{\mathrm{k}=1}^{2}{\mathrm{y}}_{\mathrm{i}\mathrm{k}}{\hspace{0.17em}}\mathrm{l}\mathrm{o}\mathrm{g}{\mathrm{p}}_{\mathrm{i}\mathrm{k}}^{\mathrm{txt}}$$

As with the image classification loss, ${\mathrm{L}}_{\mathrm{c},\mathrm{t}\mathrm{x}\mathrm{t}}$ becomes small only when the text branch assigns high probability to the correct class for each sample. Optimizing both ${\mathrm{L}}_{\mathrm{c},\mathrm{i}\mathrm{m}\mathrm{g}}$ and ${\mathrm{L}}_{\mathrm{c},\mathrm{t}\mathrm{x}\mathrm{t}}$ ensures that the shared classifier head $\mathrm{g}(\cdot)$ remains discriminative for distinguishing normal from TB cases based on either modality alone.

To explicitly couple the modalities, we used two alignment terms acting on the projected embeddings ${\mathbf{z}}_{\mathrm{i}}^{\mathrm{img}}$and ${\mathbf{z}}_{\mathrm{i}}^{\mathrm{txt}}$. First, a cosine similarity loss is used to encourage paired image–text embeddings to be close.

5 $${\mathrm{L}}_{\mathrm{c}\mathrm{o}\mathrm{s}}=\frac{1}{\mathrm{N}}\sum_{\mathrm{i}=1}^{\mathrm{N}}(1-\frac{⟨{\mathbf{z}}_{\mathrm{i}}^{\mathrm{img}},{\mathbf{z}}_{\mathrm{i}}^{\mathrm{txt}}⟩}{\parallel{\mathbf{z}}_{\mathrm{i}}^{\mathrm{img}}{\parallel}_{2}\parallel{\mathbf{z}}_{\mathrm{i}}^{\mathrm{txt}}{\parallel}_{2}})$$

Second, we adopted a supervised NT-Xent–style contrastive loss [30] defined over a mini-batch of size $\mathrm{B}$. The supervised NT-Xent contrastive term used to couple image and text embeddings operates on the ${\mathrm{L}}_{2}$-normalized outputs of the projection heads. For a mini-batch of size $\mathrm{B}$, let ${\stackrel{\sim}{\mathbf{z}}}_{\mathrm{i}}^{\mathrm{img}},{\stackrel{\sim}{\mathbf{z}}}_{\mathrm{i}}^{\mathrm{txt}}\in{\mathbb{R}}^{\mathrm{d}}$denote the normalized image and text embeddings for the i^th^ image–report pair.

The temperature-scaled contrastive loss ${\mathrm{L}}_{\mathrm{N}\mathrm{T}\mathrm{X}}\left({\uptau}\right)$ is computed as,

    

6 $$\begin{array}{l} {\mathrm L}_{\mathrm{NTX}}\left(\tau\right)=\frac1{2\mathrm B}\sum_{\mathrm i=1}^{\mathrm B}\\\lbrack-\log\frac{\exp(\widetilde{\mathbf z}_{\mathrm i}^{\mathrm{img}}\cdot\widetilde{\mathbf z}_{\mathrm i}^{\mathrm{txt}}/\tau)}{\sum_{\mathrm j=1}^{\mathrm B}\exp(\widetilde{\mathbf z}_{\mathrm i}^{\mathrm{img}}\cdot\widetilde{\mathbf z}_{\mathrm j}^{\mathrm{txt}}/\tau)}\left|-\right.\log\frac{\exp(\widetilde{\mathbf z}_{\mathrm i}^{\mathrm{txt}}\cdot\widetilde{\mathbf z}_{\mathrm i}^{\mathrm{img}}/\tau)}{\sum_{\mathrm j=1}^{\mathrm B}\exp(\widetilde{\mathbf z}_{\mathrm i}^{\mathrm{txt}}\cdot\widetilde{\mathbf z}_{\mathrm j}^{\mathrm{img}}/\tau)}\rbrack \end{array}$$

Here, ${\uptau}$ denotes the temperature hyperparameter. Although both ${\mathrm{L}}_{\mathrm{N}\mathrm{T}\mathrm{X}}$ and ${\mathrm{L}}_{\mathrm{c}\mathrm{o}\mathrm{s}}$ encourage the image and text embeddings to align, they do so in different ways. ${\mathrm{L}}_{\mathrm{N}\mathrm{T}\mathrm{X}}$is a batch‑based contrastive loss that pulls matched pairs together while pushing them apart from other samples in the minibatch, so its behavior depends on how many negatives are present and how similar they are. In contrast, ${\mathrm{L}}_{\mathrm{c}\mathrm{o}\mathrm{s}}$ applies a simple, direct alignment to each matched image–text pair, independent of the negative examples. This pairwise constraint helps stabilize training in small‑batch settings and ensures that matched pairs remain consistently similar even when batch composition varies. The overall multimodal objective combines classification and alignment as shown below:

7 $${\mathrm{L}}_{\mathrm{t}\mathrm{o}\mathrm{t}\mathrm{a}\mathrm{l}}={\mathrm{L}}_{\mathrm{c},\mathrm{i}\mathrm{m}\mathrm{g}}+{\mathrm{L}}_{\mathrm{c},\mathrm{t}\mathrm{x}\mathrm{t}}+{\mathrm{L}}_{\mathrm{c}\mathrm{o}\mathrm{s}}+{\uplambda} {\mathrm{L}}_{\mathrm{N}\mathrm{T}\mathrm{X}}\left({\uptau}\right)$$

Here, ${\uplambda}$ serves as a weighting hyperparameter for the contrastive loss component, controlling the level of emphasis the model places on aligning the image and text embeddings. For the unimodal baseline, the loss objective simplifies to ${\mathrm{L}}_{\mathrm{t}\mathrm{o}\mathrm{t}\mathrm{a}\mathrm{l}}={\mathrm{L}}_{\mathrm{c},\mathrm{i}\mathrm{m}\mathrm{g}}$.

### Training and Model Checkpointing

The unimodal and multimodal models were optimized with AdamW using a learning rate of $5\times{10}^{-5}$, weight decay of ${10}^{-4}$, mini-batch size $\left(\mathrm{B}\right)$of 64, and a maximum of 64 epochs. During multimodal training, the vision encoder, the image projection layer, the text projection layer, and the shared classification head were updated jointly under ${\mathrm{L}}_{\mathrm{t}\mathrm{o}\mathrm{t}\mathrm{a}\mathrm{l}}$, while the CXR-BERT text encoder weights remained frozen, to mitigate overfitting given limited training data. In unimodal training, only the image encoder and classification head receive gradients from ${\mathrm{L}}_{\mathrm{c},\mathrm{i}\mathrm{m}\mathrm{g}}$. At the end of each epoch, the total loss ${\mathrm{L}}_{\mathrm{t}\mathrm{o}\mathrm{t}\mathrm{a}\mathrm{l}}$ is computed on the validation split to monitor how well the model optimizes the full multimodal objective. However, model checkpoints were stored whenever the validation MCC increased, and the final model used for evaluation is the checkpoint with the highest validation MCC, not necessarily the last epoch or the lowest loss. MCC is used as the selection metric because it is known to be robust under class imbalance and to provide a balanced summary of binary classification performance in biomedical applications. [31] Early stopping is applied if the validation Matthews correlation coefficient (MCC) fails to improve for 10 consecutive epochs.

### Hyperparameter Optimization

Hyperparameter tuning targets the alignment terms $({\uplambda},{\uptau})$, which regulate how strongly the model aligns image and text embeddings. Optimizing $({\uplambda},{\uptau})$ is critical in multimodal learning because the parameters control the trade-off between within-modality discrimination and cross-modal alignment. If ${\uplambda}$ is too small, the contrastive term contributes negligibly to ${\mathrm{L}}_{\mathrm{t}\mathrm{o}\mathrm{t}\mathrm{a}\mathrm{l}}$; image–text pairs remain weakly aligned, and the image encoder behaves similarly to a purely unimodal image-only classifier, limiting any benefit from the clinical text. Conversely, if ${\uplambda}$ is too large, the optimization over-emphasizes matching image and text embeddings at the expense of separating normal from TB cases; this can yield tightly aligned but poorly discriminative representations, an effect also noted when supervised contrastive objectives are over-weighted relative to cross-entropy in other domains. [32] The temperature ${\uptau}$ plays a complementary role by controlling the sharpness of the contrastive distribution. Small ${\uptau}$ magnifies differences between similarity logits and heavily up-weights the hardest negatives, which can accelerate representation sharpening but also makes training unstable for small, noisy, or class-imbalanced medical batches. Larger ${\uptau}$ smooths the distribution over negatives and produces more conservative gradients, but overly large values dilute the contrastive signal and weaken cross-modal alignment. Prior analyses of NT-Xent and supervised contrastive learning have shown that performance and stability are highly sensitive to ${\uptau}$. [33] Similar sensitivity of the ${\uptau}$ parameter has been documented in CLIP-style vision–language models, where it directly governs the strength of penalties on negative pairs. [34] Because our internal Shenzhen CXR dataset is small sized, there is no analytically optimal choice of $({\uplambda},{\uptau})$; the effective balance between leveraging text as privileged information and preserving robust image-based discrimination is inherently data-dependent. Therefore, we perform an explicit grid search over ${\uplambda}\in\{0.1,\dots,0.9\}$ and ${\uptau}\in\{0.05,\dots,0.09\}$, training a multimodal model for each pair, and selecting the checkpoint with the highest validation MCC. The checkpoint whose validation MCC is maximal within each regime (unimodal, multimodal-raw, multimodal-structured) is then evaluated on internal and external test sets.

### Performance Evaluation

All quantitative performance measures were derived from the true positives (TP), false positives (FP), false negatives (FN), and true negatives (TN), together with the model’s predicted probabilities for TB for the area under the receiver-operating-characteristic (ROC) curve (AUC). Because screening for pulmonary TB is a safety-critical task, we report a panel of complementary metrics that separately characterize sensitivity, specificity, predictive values, and overall discriminative ability. Sensitivity (Recall) quantifies the proportion of truly TB radiographs that the model correctly identifies and is therefore directly related to the ability of the system to avoid missed TB cases. It is computed as the fraction of TB-positive examinations that the model classifies as positive among all TB-positive examinations.

8 $$\mathrm{S}\mathrm{e}\mathrm{n}\mathrm{s}\mathrm{i}\mathrm{t}\mathrm{i}\mathrm{v}\mathrm{i}\mathrm{t}\mathrm{y}=\frac{\mathrm{T}\mathrm{P}}{\mathrm{T}\mathrm{P}+\mathrm{F}\mathrm{N}}$$

Specificity measures how well the model avoids incorrectly labeling normal radiographs as manifesting TB, capturing its tendency to generate false alarms. It is computed as the proportion of truly normal examinations correctly identified as normal among all normal examinations.

  

9 $$\mathrm{S}\mathrm{p}\mathrm{e}\mathrm{c}\mathrm{i}\mathrm{f}\mathrm{i}\mathrm{c}\mathrm{i}\mathrm{t}\mathrm{y}=\frac{\mathrm{T}\mathrm{N}}{\mathrm{T}\mathrm{N}+\mathrm{F}\mathrm{P}}$$

Balanced accuracy provides a single measure that accounts for both sensitivity and specificity, which is important when class distributions are skewed. It averages sensitivity and specificity so that performance on the TB-positive class and the normal class contribute equally.

10 $$\mathrm{B}\mathrm{a}\mathrm{l}\mathrm{a}\mathrm{n}\mathrm{c}\mathrm{e}\mathrm{d}\mathrm{A}\mathrm{c}\mathrm{c}\mathrm{u}\mathrm{r}\mathrm{a}\mathrm{c}\mathrm{y}=\frac{1}{2}(\mathrm{S}\mathrm{e}\mathrm{n}\mathrm{s}\mathrm{i}\mathrm{t}\mathrm{i}\mathrm{v}\mathrm{i}\mathrm{t}\mathrm{y}+\mathrm{S}\mathrm{p}\mathrm{e}\mathrm{c}\mathrm{i}\mathrm{f}\mathrm{i}\mathrm{c}\mathrm{i}\mathrm{t}\mathrm{y})$$

Precision (positive predictive value (PPV)) reflects the reliability of a positive test result, i.e., the proportion of model-positive predictions that correspond to truly TB radiographs. It is expressed as the fraction of correctly identified TB-positive cases among all cases flagged as TB-positive by the model.

11 $$\mathrm{P}\mathrm{r}\mathrm{e}\mathrm{c}\mathrm{i}\mathrm{s}\mathrm{i}\mathrm{o}\mathrm{n}=\frac{\mathrm{T}\mathrm{P}}{\mathrm{T}\mathrm{P}+\mathrm{F}\mathrm{P}}$$

 

Negative predictive value (NPV) reflects the reliability of a negative prediction, capturing how often a CXR predicted as normal is truly free of TB-consistent manifestations. It computes the proportion of correctly identified normal cases among all cases predicted as normal.

12 $$\mathrm{N}\mathrm{P}\mathrm{V}=\frac{\mathrm{T}\mathrm{N}}{\mathrm{T}\mathrm{N}+\mathrm{F}\mathrm{N}}$$

The F1 score (F1) summarizes the trade-off between sensitivity and precision in a single harmonic-mean measure, which is useful when both missed detections and false alarms are clinically important. The score increases only when both sensitivity and precision improve.

13 $$\mathrm{F}1=\frac{2\text{ }\mathrm{T}\mathrm{P}}{2\text{ }\mathrm{T}\mathrm{P}+\mathrm{F}\mathrm{P}+\mathrm{F}\mathrm{N}}$$

 

Youden’s J (J) index provides an interpretable summary of screening performance, with values ranging from 0 (no better than chance) to 1 (perfect separation). J-index combines sensitivity and specificity into a single index that quantifies the maximum vertical distance between the ROC curve and the diagonal line of no discrimination.

14 $$\mathrm{J}=\mathrm{S}\mathrm{e}\mathrm{n}\mathrm{s}+\mathrm{S}\mathrm{p}\mathrm{e}\mathrm{c}-1$$

MCC is a balanced correlation-like measure that incorporates all four confusion-matrix entries and is particularly informative under class imbalance. MCC normalizes the difference between concordant terms ($\mathrm{T}\mathrm{P}\cdot\mathrm{T}\mathrm{N}$) and discordant terms ($\mathrm{F}\mathrm{P}\cdot\mathrm{F}\mathrm{N}$) by all possible combinations, yielding values in $[-\mathrm{1,1}]$ where 1 indicates perfect predictions, and 0 corresponds to random performance.

      

15 $$\mathrm{M}\mathrm{C}\mathrm{C}=\frac{\mathrm{T}\mathrm{P}\cdot\mathrm{T}\mathrm{N}-\mathrm{F}\mathrm{P}\cdot\mathrm{F}\mathrm{N}}{\sqrt{(\mathrm{T}\mathrm{P}+\mathrm{F}\mathrm{P})(\mathrm{T}\mathrm{P}+\mathrm{F}\mathrm{N})(\mathrm{T}\mathrm{N}+\mathrm{F}\mathrm{P})(\mathrm{T}\mathrm{N}+\mathrm{F}\mathrm{N})}}$$

AUC characterizes the global discriminative ability of the model by integrating the ROC curve constructed from predicted TB probabilities across all possible decision thresholds. It is computed numerically from the ranked list of probabilities and corresponding true labels and can be interpreted as the probability that a randomly chosen TB-positive examination receives a higher predicted probability than a randomly chosen normal examination.

To compare the relative performance gains of the multimodal variants over the unimodal baseline, we define delta-performance metrics for both MCC and Youden’s J. Specifically, we compute the absolute difference between respective multimodal and unimodal models.

16 $${\Delta}{\mathrm{M}\mathrm{C}\mathrm{C}}_{\mathrm{raw}}={|\mathrm{M}\mathrm{C}\mathrm{C}}_{\mathrm{mm-raw}}-{\mathrm{M}\mathrm{C}\mathrm{C}}_{\mathrm{uni}}|$$

17 $${\Delta}{\mathrm{M}\mathrm{C}\mathrm{C}}_{\mathrm{str}}={|\mathrm{M}\mathrm{C}\mathrm{C}}_{\mathrm{mm-str}}-{\mathrm{M}\mathrm{C}\mathrm{C}}_{\mathrm{uni}}|$$

18 $${\Delta}{\mathrm{J}}_{\mathrm{raw}}={|\mathrm{J}}_{\mathrm{mm-raw}}-{\mathrm{J}}_{\mathrm{uni}}|$$

19 $${\Delta}{\mathrm{J}}_{\mathrm{str}}=|{\mathrm{J}}_{\mathrm{mm-str}}-{\mathrm{J}}_{\mathrm{uni}}|$$

Here, ${\mathrm{M}\mathrm{C}\mathrm{C}}_{\mathrm{uni}}$ and ${\mathrm{J}}_{\mathrm{uni}}$ denote the MCC and Youden’s J index of the unimodal model, whereas ${\mathrm{M}\mathrm{C}\mathrm{C}}_{\mathrm{mm-raw}}$, ${\mathrm{M}\mathrm{C}\mathrm{C}}_{\mathrm{mm-str}}$, ${\mathrm{J}}_{\mathrm{mm-raw}}$, and ${\mathrm{J}}_{\mathrm{mm-str}}$ denote the corresponding metrics for the multimodal-raw and multimodal-structured models, respectively. Positive values of ${\Delta}{\mathrm{M}\mathrm{C}\mathrm{C}}_{\mathrm{raw}}$, ${\Delta}{\mathrm{M}\mathrm{C}\mathrm{C}}_{\mathrm{str}}$, ${\Delta}{\mathrm{J}}_{\mathrm{raw}}$, and ${\Delta}{\mathrm{J}}_{\mathrm{str}}$ indicate improvement over the unimodal baseline, and comparing the magnitudes of these deltas allows us to determine which multimodal variant yields the larger gain and screening effectiveness relative to the unimodal baseline. We emphasize validation‑driven model selection and external validation as the primary safeguards against over‑interpreting fluctuations on the internal test set. The explicit $({\uplambda},{\uptau})$ grid search is computationally intensive but reduces sensitivity to arbitrary alignment‑weight and temperature choices in a small‑data setting. Accordingly, our conclusions rely on (i) selecting the best‑validation checkpoint within each training regime, (ii) observing improvements that repeat across three independent external cohorts, and (iii) reporting performance differences of meaningful magnitude using both standard metrics and delta‑measures (${\Delta}\mathrm{M}\mathrm{C}\mathrm{C}$ and ${\Delta}\mathrm{J}$).

### Computational Tools

Experiments were implemented in Python 3.11.11. The unimodal and multimodal models were developed with PyTorch 2.7.0 and trained using NVIDIA CUDA 12.6 on an A100 GPU. The CXR‑BERT architecture was implemented with Transformers 4.45.2 to handle textual report preprocessing. Model performance was evaluated with scikit‑learn 1.7.1, while Numpy 2.2.5 supported numerical operations. Image preprocessing, CLAHE augmentation, normalization, and tensor conversion were performed using Albumentations 2.0.8.

## Results

### Quantitative performance

Multimodal learning consistently improved downstream image-only prediction relative to the unimodal image-only baseline across all datasets, with the multimodal-structured variant providing the largest and most stable gains, as shown in Table 3. The optimal balance favored slightly weaker, higher-temperature alignment for raw notes (𝜆,𝜏)=(0.5,0.07), consistent with the need to avoid over-constraining the image encoder to noisy narrative cues, whereas structured reports supported a marginally stronger, lower-temperature alignment (𝜆,𝜏)=(0.6,0.05), reflecting their cleaner, more anatomically anchored semantics.

**Table 3:** Unimodal and multimodal classification performance with internal and external test sets

Modality	(λ, τ)	TP	FP	FN	TN	Bal. Acc.	Sens.	Spec.	Prec.	NPV	F1	MCC	J	Δ MCC	Δ J
Shenzhen CXR															
Unimodal		28	5	6	28	0.8360	0.8235	0.8485	0.8485	0.8235	0.8358	0.6720	0.6720		
Multi-raw	(0.5,0.07)	28	1	6	32	0.8966	0.8235	0.9697	0.9655	0.8421	0.8889	0.8004	0.7932	0.1284	0.1212
Multi-structured	(0.6,0.05)	30	2	4	31	0.9109	0.8824	0.9394	0.9375	0.8857	0.9091	0.8225	0.8218	0.1505	0.1498
MC CXR															
Unimodal		58	77	0	3	0.5188	1.0000	0.0375	0.4296	1.0000	0.6010	0.1269	0.0376		
Multi-raw	(0.5,0.07)	16	1	42	79	0.6317	0.2758	0.9875	0.9412	0.6529	0.4267	0.3955	0.2633	0.2686	0.2257
Multi-structured	(0.6,0.05)	28	3	30	77	0.7227	0.4828	0.9625	0.9032	0.7196	0.6292	0.5266	0.4453	0.3997	0.4077
TBX11K CXR															
Unimodal		771	2008	28	1792	0.7183	0.9650	0.4716	0.2774	0.9846	0.4310	0.3382	0.4366		
Multi-raw	(0.5,0.07)	537	207	262	3593	0.8088	0.6721	0.9455	0.7218	0.9320	0.6960	0.6355	0.4809	0.2973	0.0443
Multi-structured	(0.6,0.05)	586	42	213	3758	0.8612	0.7334	0.9889	0.9331	0.9464	0.8213	0.7971	0.7223	0.4589	0.2857

Using the model checkpoints saved using these optimized hyperparameters that maximized validation MCC, improvements were evident in all metrics, and these improvements were particularly pronounced under external validation. On the internal Shenzhen CXR test set, the unimodal baseline achieved balanced accuracy of 0.8360, MCC of 0.6720, and J of 0.6720. Multimodal-raw variant increased balanced accuracy to 0.8966 and MCC to 0.8004, corresponding to an absolute ${\Delta}{\mathrm{M}\mathrm{C}\mathrm{C}}_{\mathrm{raw}}$ of 0.1284 and ${\Delta}{\mathrm{J}}_{\mathrm{raw}}$ of 0.1212 relative to the unimodal baseline. Multimodal-structured variant further improved balanced accuracy to 0.9109, MCC to 0.8225, and J to 0.8218, yielding ${\Delta}{\mathrm{M}\mathrm{C}\mathrm{C}}_{\mathrm{str}}$ = 0.1505 and ${\Delta}{\mathrm{J}}_{\mathrm{str}}$ = 0.1498. These gains arose from simultaneous preservation or improvement of both sensitivity (0.8235 → 0.8235 → 0.8824) and specificity (0.8485 → 0.9697 → 0.9394), indicating that multimodal supervision sharpened the decision boundary without sacrificing recall for TB-consistent abnormalities.

Under external validation on the MC CXR dataset, the unimodal baseline showed strong sensitivity (1.0000) but extremely poor specificity (0.0375), yielding balanced accuracy of 0.5188, MCC of 0.1269, and J of 0.0376. In other words, the unimodal model labeled nearly all CXRs as TB-positive (TP 58, FP 77, FN 0, TN 3), a pattern that would be clinically unsustainable. Multimodal-raw variant notably improved specificity to 0.9875 and balanced accuracy to 0.6317, but at the expense of reduced sensitivity (0.2758); nevertheless, MCC increased to 0.3955 and J to 0.2633 (${\Delta}{\mathrm{M}\mathrm{C}\mathrm{C}}_{\mathrm{raw}}$= 0.2686, ${\Delta}{\mathrm{J}}_{\mathrm{raw}}$ = 0.2257). Multimodal-structured model provided a more clinically balanced trade-off, achieving balanced accuracy of 0.7227, sensitivity of 0.4828, and specificity of 0.9625, with MCC of 0.5266 and J of 0.4453 (${\Delta}{\mathrm{M}\mathrm{C}\mathrm{C}}_{\mathrm{str}}$ = 0.3997, ${\Delta}{\mathrm{J}}_{\mathrm{str}}$ = 0.4077). Relative to the unimodal baseline, the multimodal-structured variant reduced false positives from 77 to 3 (≈96% reduction) while retaining 28 true positives (vs. 58), yielding the highest overall discriminative strength.

On the external TBX11K test cohort, the unimodal baseline again operated in a highly sensitive but poorly specific regime (TP 771, FP 2008, FN 28, TN 1792), with balanced accuracy of 0.7183, MCC of 0.3382, and J of 0.4366. Multimodal learning with raw notes improved balanced accuracy to 0.8088 and MCC to 0.6355 (${\Delta}{\mathrm{M}\mathrm{C}\mathrm{C}}_{\mathrm{raw}}$ = 0.2973), with sensitivity of 0.6721 and specificity of 0.9455. The corresponding J value increased modestly to 0.4809 (${\Delta}{\mathrm{J}}_{\mathrm{raw}}$ = 0.0443), reflecting an improved but still imbalanced operating point.

The multimodal-structured variant produced the strongest performance, with balanced accuracy of 0.8612, MCC of 0.7971, and J of 0.7223 (${\Delta}{\mathrm{M}\mathrm{C}\mathrm{C}}_{\mathrm{str}}$ = 0.4589, ${\Delta}{\mathrm{J}}_{\mathrm{str}}$ = 0.2857). Importantly, this model reduced false positives from 2008 to 42 (>97% reduction) while correctly identifying 586 of 799 TB-positive CXRs (sensitivity of 0.7334) and maintaining very high specificity (0.9889). Multimodal learning with structured notes converted the prediction head from a high-sensitivity, low-precision screener into a substantially more balanced classifier.

**Fig. 2:**
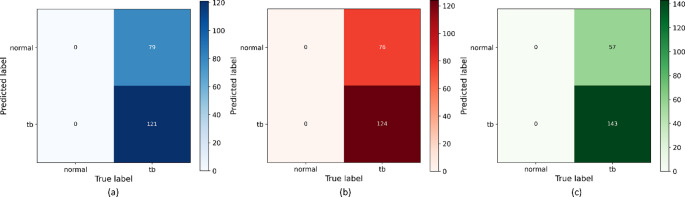
Confusion matrices when evaluated with the external NIAID TB Portals cohort. (**a**) Unimodal, (**b**) Multimodal-raw, (**c**) Multimodal-structured

The NIAID TB Portals CXR cohort contains only confirmed TB cases with diverse drug-resistance phenotypes and no normal controls; hence, balanced accuracy, specificity, and related measures cannot be estimated. Therefore, our evaluation focuses on TB recall and its complement, the false-negative rate (FNR), which are the most relevant quantities in a case-only (TB) population. We observed that the unimodal baseline achieved a sensitivity of 0.6050 (TP = 121, FN = 79; FNR = 0.3800), among 200 confirmed TB cases (Table 4; Fig. 2(a)). The multimodal-raw variant yielded a modest improvement, with sensitivity increasing to 0.6200 (TP = 124, FN = 76; FNR = 0.3800), corresponding to a 1.5% gain in sensitivity relative to the unimodal model (Table 4; Fig. 2(b)). In contrast, the multimodal-structured model demonstrated a substantially larger benefit in this setting. Sensitivity of this model increased to 0.7150 (TP = 143, FN = 57; FNR = 0.2850) (Table 4; Fig. 2(c)), representing an absolute gain of 11% gain in sensitivity relative to the unimodal baseline and 9.5% sensitivity gain over the multimodal-raw variant. This corresponds to a relative reduction in FNR of approximately 28% and 25% compared with the unimodal baseline (0.3950 → 0.2850) and multimodal-raw model (0.3800 → 0.2850), respectively.

**Table 4:** Unimodal and multimodal classification performance with the external NIAID TB Portals cohort

Modality	(λ, τ)	TP	FN	Sens.	FNR
Unimodal		121	79	0.6050	0.3950
Multi-raw	(0.5,0.07)	124	76	0.6200	0.3800
Multi-structured	(0.6,0.05)	**143**	**57**	**0.7150**	**0.2850**

Bold numerical values along columns denote superior performance

**Fig. 3:**
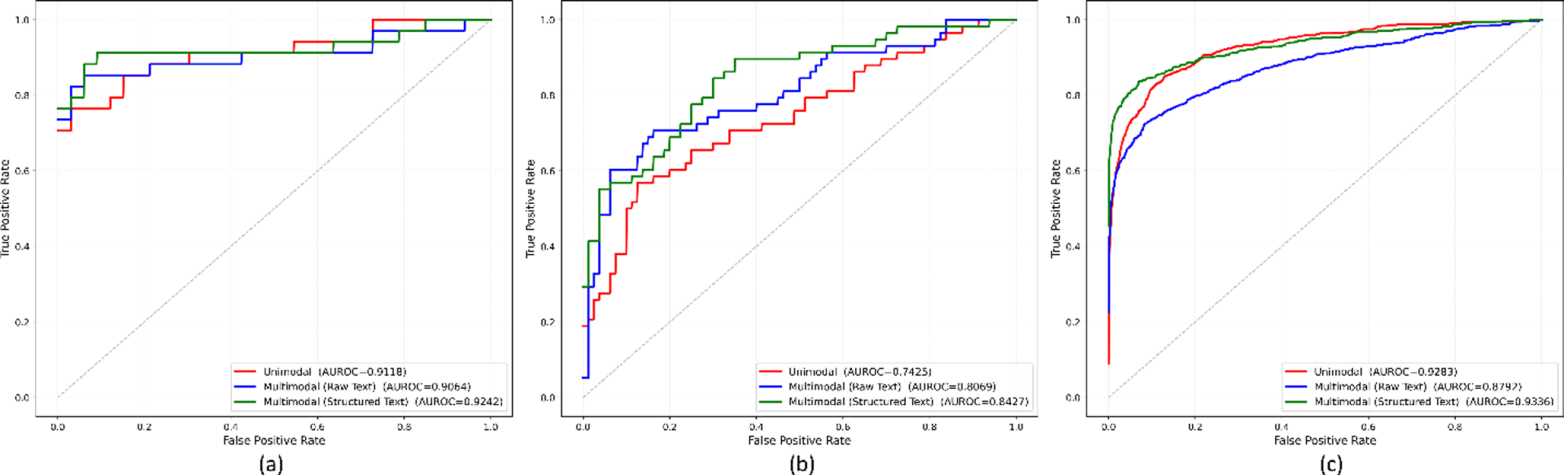
Unimodal and multimodal ROC comparison for internal and external test cohorts. (**a**) Shenzhen CXR, (**b**) MC CXR, (**c**) TBX11K CXR

The ROC curves generated for the internal Shenzhen test and the external MC CXR and TBX11K test sets as illustrated in Fig. 3 showed a consistent right-upward shift of the multimodal-structured model compared with the unimodal baseline. The gain in AUC is largest on the external MC test (0.8427 vs. 0.7425), and intermediate on the external TBX11K cohort (0.9336 vs. 0.9283) and internal Shenzhen test set (0.9242 vs. 0.9118). In particular, the curve for the multimodal-structured variant separated most clearly from the unimodal curve at higher specificity regions, consistent with the large reductions in false positives as observed in Table 3. In summary, these results demonstrate that multimodal learning yields a more discriminative image-only head during inference, with the most substantial gains seen under external validation, and with the multimodal-structured learning, notably outperforming those trained with raw notes across metrics.

### Representation and Explainability

**Fig. 4:**
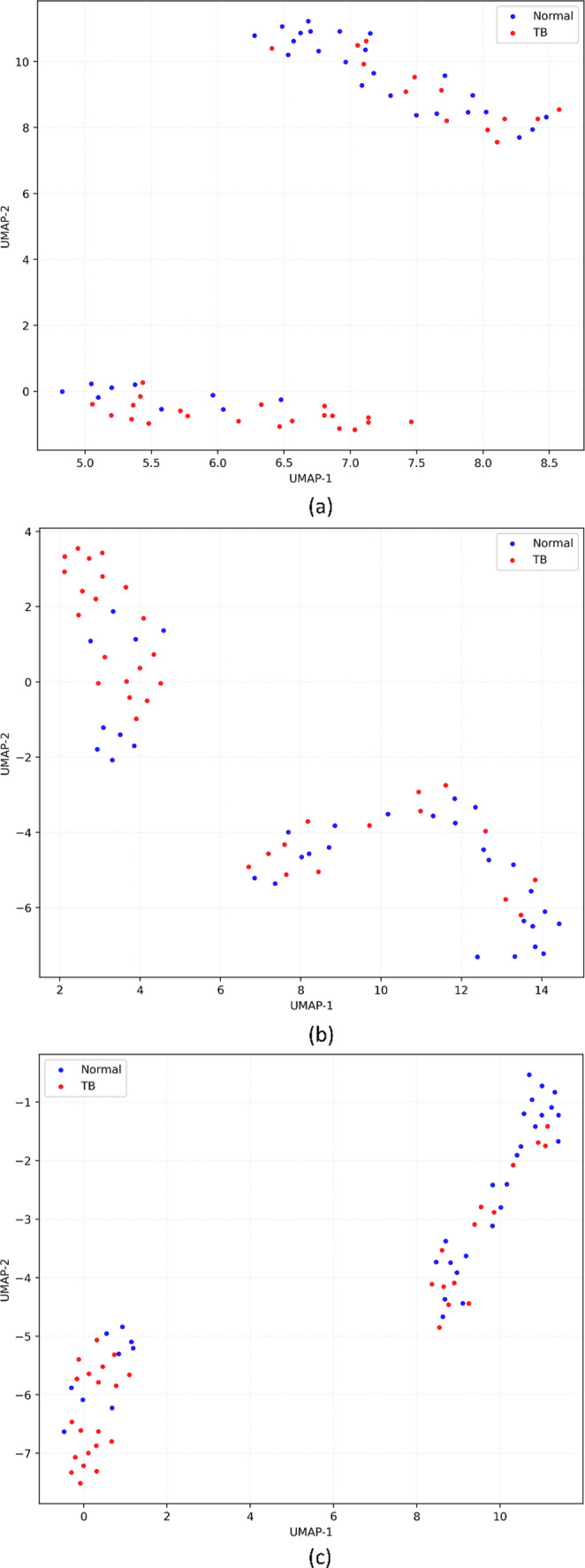
UMAP projection of vision features extracted from the deepest convolutional layer using the internal Shenzhen CXR test cohort. Each point corresponds to one test image; colors denote the ground-truth class (Normal vs. TB). UMAP preserves local neighborhood structure in the learned feature space (qualitative visualization only); increased separation and tighter within-class grouping indicate that the corresponding training strategy yielded more class-consistent representations. (**a**) Unimodal, (**b**) Multimodal-raw, (**c**) Multimodal-structured

Figure 4 shows the UMAP projections of the features extracted from the deepest convolutional layer of the vision encoder using the internal Shenzhen CXR test cohort, illustrating how different learning methods reshaped the representation geometry. The unimodal baseline showed partially overlapping clusters of normal and TB-positive CXRs, with a sizeable region of mixed points. In contrast, the multimodal-structured variant exhibited tighter within-class groupings and clearer interclass margins, particularly for the TB-positive cluster, indicating that the joint image–text supervision has encouraged the image encoder to map TB-consistent patterns into a more coherent region of feature space. Under external testing, similar patterns were observed.

**Fig. 5:**
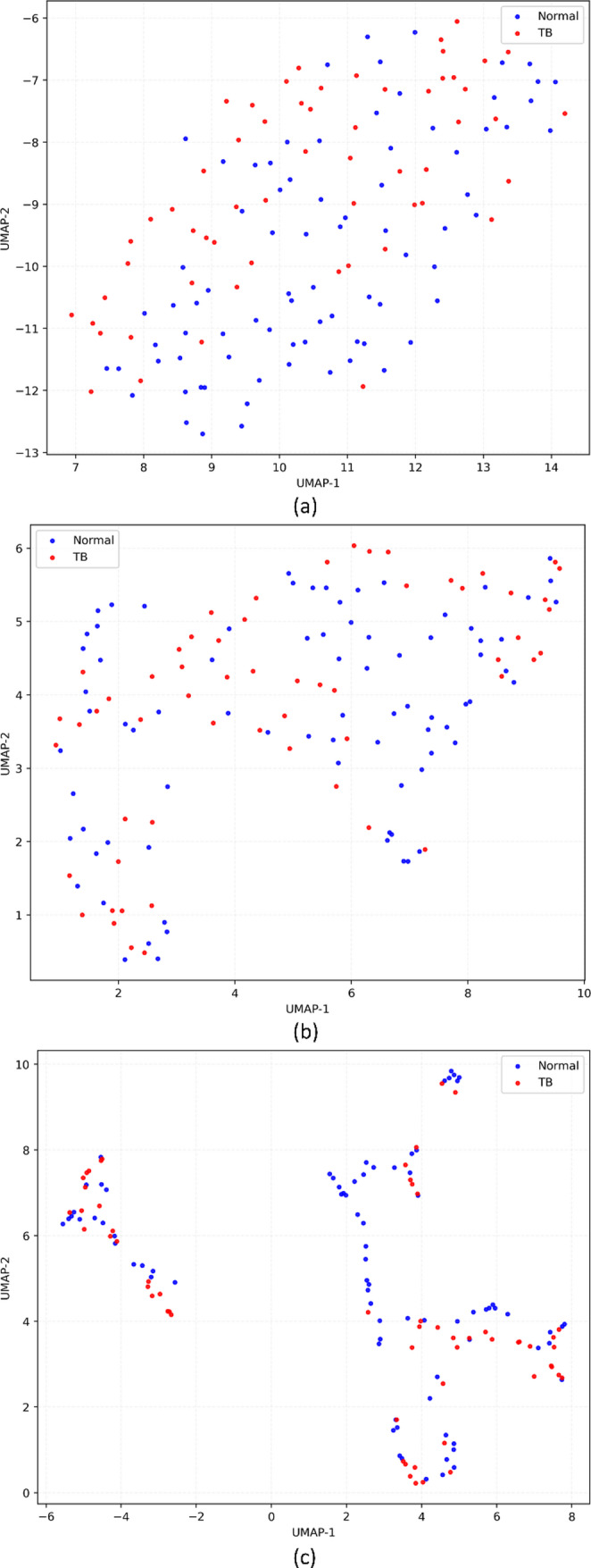
UMAP projection of vision features extracted from the deepest convolutional layer using the external Montgomery County (MC) CXR test cohort. Each point corresponds to one test image; colors denote the ground-truth class (Normal vs. TB). The plots are qualitative and intended to visualize how privileged text supervision reshapes the geometry of the image embedding space under domain shift. (**a**) Unimodal, (**b**) Multimodal-raw, (**c**) Multimodal-structured

On the embeddings extracted using the MC test cohort as shown in Fig. 5, unimodal features displayed substantial overlap between classes, consistent with the model’s tendency to over-predict TB. The multimodal-raw model partially separated normal from TB-positive cases, but many points remained in an intermediate band. The multimodal-structured model showed the clearest bifurcation, with normal and TB CXRs forming compact clusters, suggesting that multimodal structured supervision has imparted robust, transferable semantics despite differences.

**Fig. 6:**
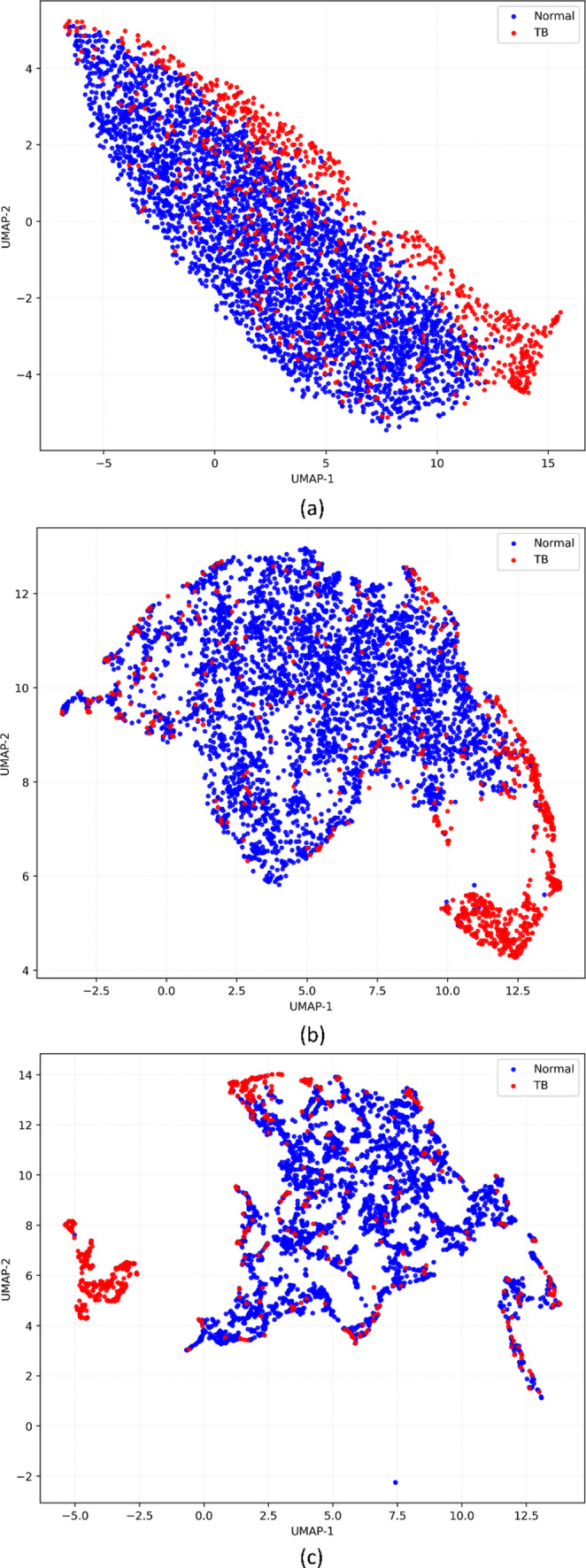
UMAP projection of vision features extracted from the deepest convolutional layer using the external TBX11K CXR test cohort. Each point corresponds to one test image; colors denote the ground-truth class (Normal vs. TB). Clearer inter-class margins and reduced overlap indicate more discriminative image representations; UMAP is used here for qualitative visualization of representation structure and not as a statistical test. (**a**) Unimodal, (**b**) Multimodal-raw, (**c**) Multimodal-structured

On the external TBX11K CXR test, where the distribution is more heterogeneous, and the set is larger, the unimodal embeddings as shown in Fig. 6 again showed diffuse intermingling, whereas the multimodal-structured embeddings demonstrated both tighter TB clusters and a greater margin from the normal cluster, aligning with the large ${\Delta}{\mathrm{M}\mathrm{C}\mathrm{C}}_{\mathrm{str}}$ observed in the quantitative results in Table 3.

**Fig. 7:**
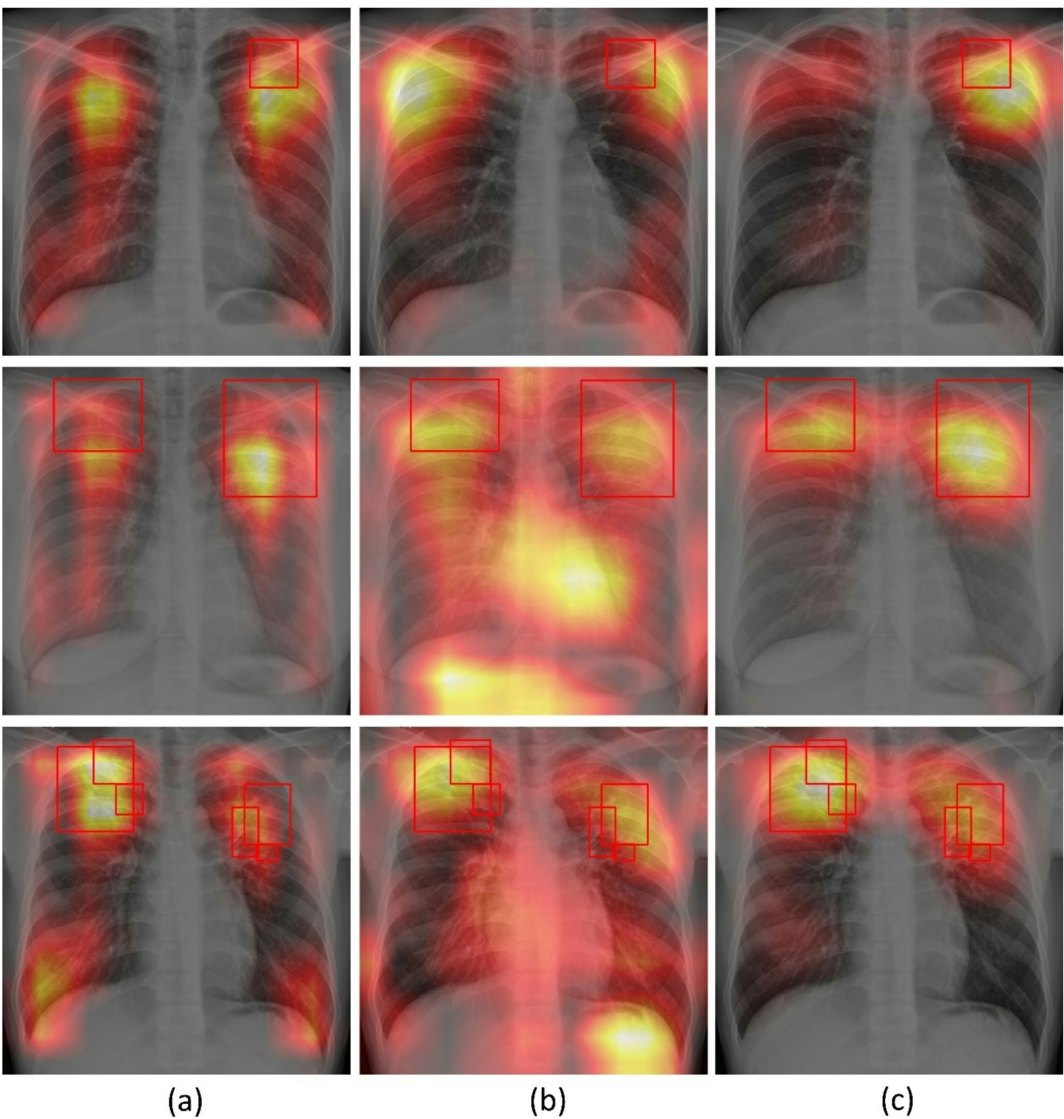
Grad-CAM heatmap activations from the deepest convolutional layer of the vision encoder using sample CXRs (*n* = 3) from the internal Shenzhen CXR test. Warmer colors indicate higher contribution to the predicted class score. (**a**) Unimodal, (**b**) Multimodal-raw, (**c**) Multimodal-structured. Red bounding boxes denote expert-annotated TB lesions

**Fig. 8:**
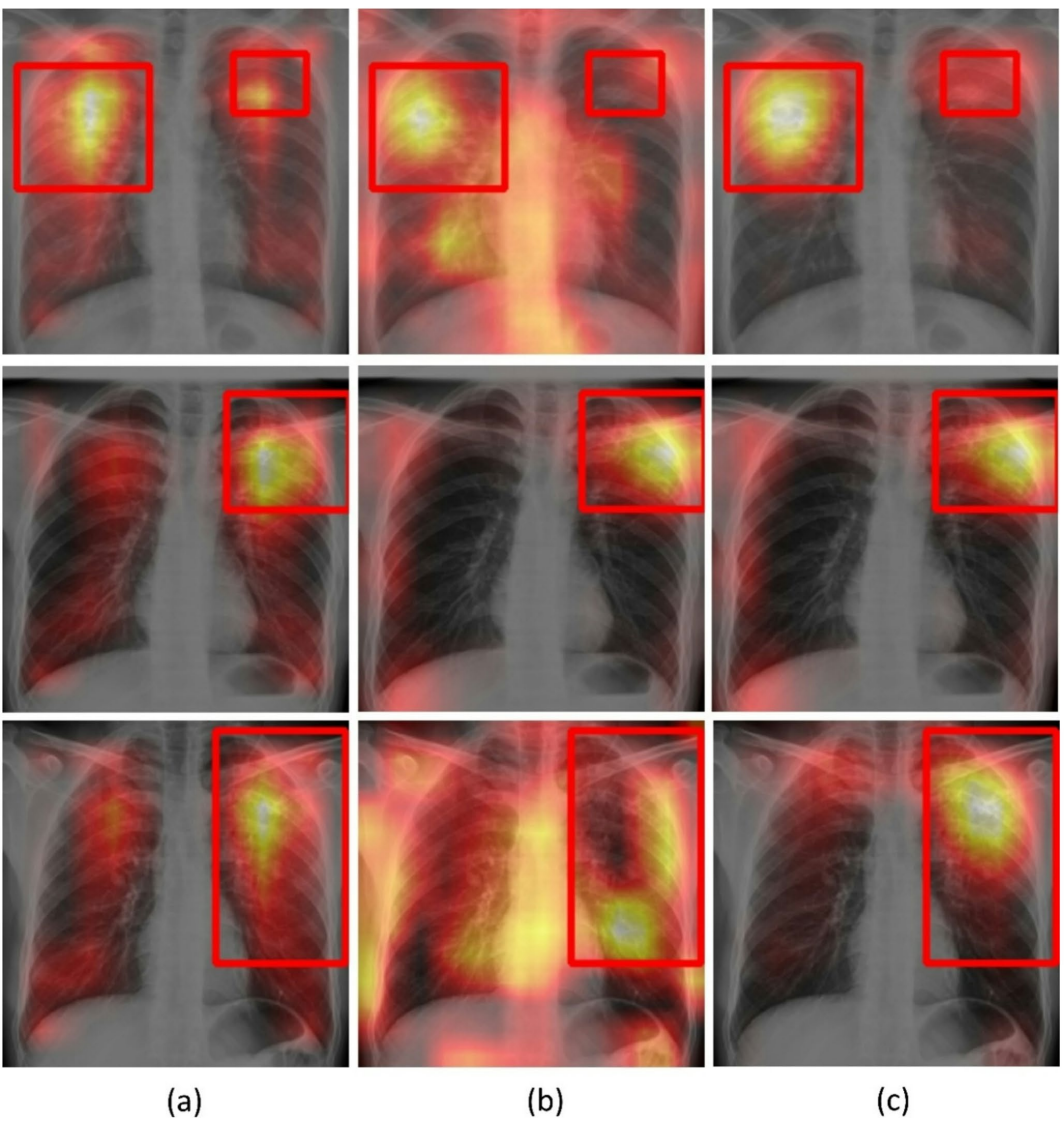
Grad-CAM heatmap activations from the deepest convolutional layer of the vision encoder using sample CXRs (*n* = 3) from the external TBX11K CXR test. Warmer colors indicate a higher contribution to the predicted class score. (**a**) Unimodal, (**b**) Multimodal-raw, (**c**) Multimodal-structured. Red bounding boxes denote expert-annotated TB lesions

Grad-CAM visualizations provide insight into changes in spatial attention due to training with text over the unimodal baseline. On the internal Shenzhen CXR test cohort (Fig. 7) and external TBX11K CXR test (Fig. 8), where ground truth lesion bounding boxes are available, the unimodal model frequently produced broad, off-target activations that extended beyond annotated lesions. In contrast, the multimodal-structured model showed focused and anatomically plausible attention, with heatmaps concentrating on cavitary, consolidative, or nodular regions marked by radiologists, and less attention on extrapulmonary regions.

**Fig. 9:**
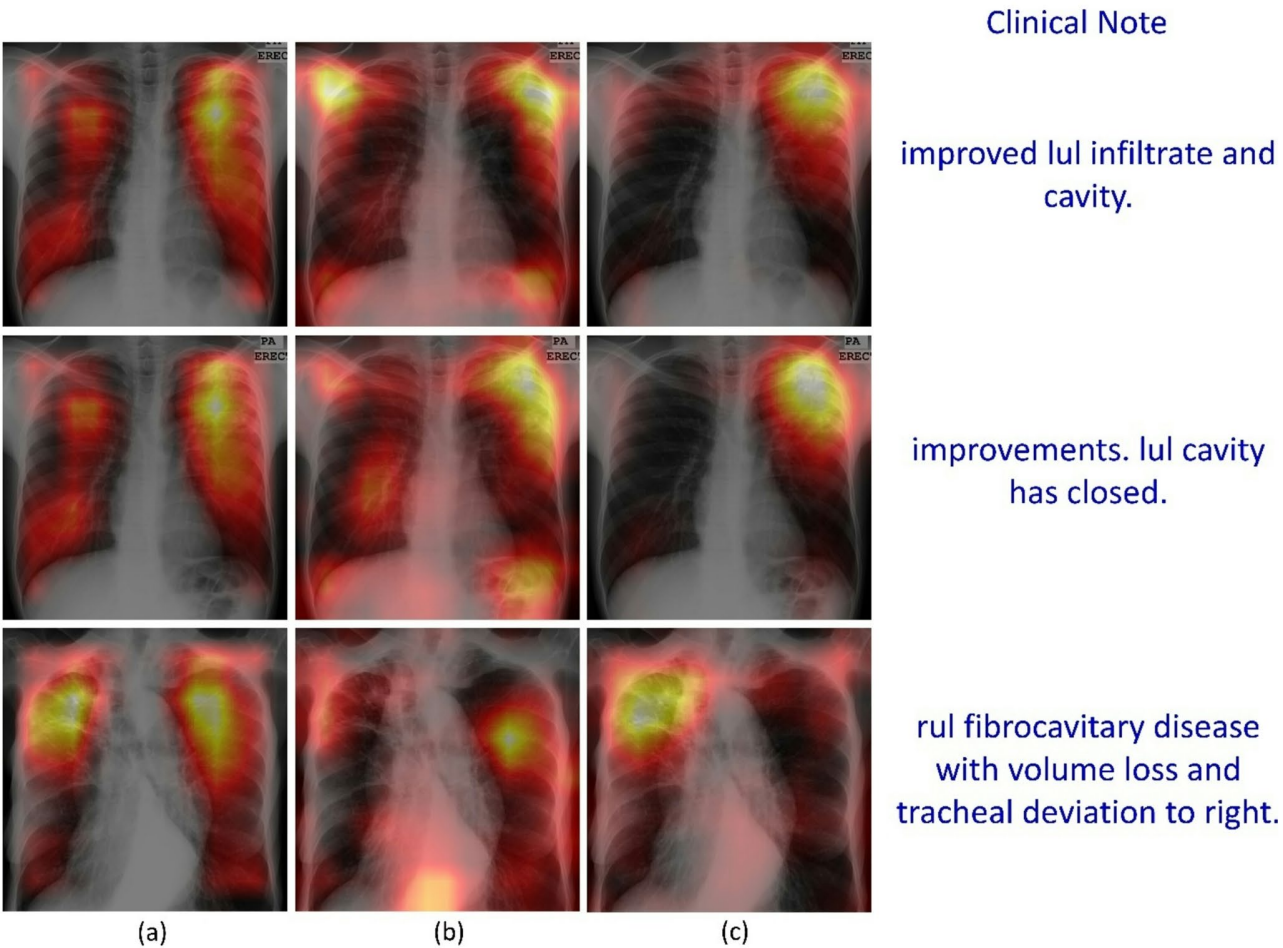
Grad-CAM heatmap activations from the deepest convolutional layer of the vision encoder using sample CXRs (*n* = 3) from the external MC CXR test. Warmer colors indicate a higher contribution to the predicted class score. (**a**) Unimodal, (**b**) Multimodal-raw, (**c**) Multimodal-structured. The corresponding raw clinical notes (shown in blue) are provided as a qualitative reference because lesion bounding boxes are not available for MC

For the external MC CXR test, explicit lesion boxes are not available, but raw clinical notes offered a surrogate reference. As shown in Fig. 9, the raw notes for sample test CXR images read “improved lul infiltrate and cavity” (1st row), “improvements, lul cavity has closed” (2nd row), and “rul fibrocavitary disease with volume loss and tracheal deviation to right” (3rd row). Here, LUL and RUL denote left or right upper lobes, respectively. The first clinical note described left upper lobe cavitary disease, the unimodal model spread attention across both lungs and upper mediastinum, whereas the multimodal-raw model shifted attention toward the left upper zone but still showed substantial off-target activation. The multimodal-structured model tended to generate Grad-CAM heatmaps that were more tightly concentrated in the reported left upper lobar regions and less influenced by non-parenchymal structures. In the 2nd note, both the unimodal and multimodal-raw models show broad bilateral activation, whereas the multimodal-structured model yields a more localized focus in the left upper zone, consistent with the clinical note mentioning cavity presence in the left upper lobe. In the 3rd note, the unimodal and multimodal-raw models attend to both the lungs, while the multimodal-structured model places its highest activation in the right apical and upper-lobe parenchyma, reflecting a distilled emphasis on right upper lobe fibrocavitary disease as mentioned in the clinical note. These qualitative patterns support the conclusion that multimodal learning with structured reports guided the vision encoder toward more clinically meaningful and text-consistent saliency patterns. We provide an expanded set of Grad-CAM visualizations across internal and external cohorts in Supplementary Figures (S1 – S3) to further assess the qualitative consistency of these saliency patterns beyond the representative examples shown in Figures (7, 8 and 9). The additional examples show that the multimodal-structured model consistently produces more lesion-proximal and fewer off-target activations compared with unimodal and multimodal-raw variants.

## Discussion

### Key Findings

Our study evaluated the efficacy of an image-text alignment-regularized inference strategy: using image metadata and brief clinical text as privileged information following Vapnik’s LUPI framework during training to investigate whether the vision encoder can learn semantically meaningful and robust features during image-only inference. The combined quantitative and qualitative findings from this study demonstrate benefits to the image-head that are achieved due to privileged supervision during multimodal training. Radiology text encodes lesion types, disease status/subtype qualifiers, and anatomical locations that are not directly specified in a binary image label. Aligning image embeddings with text embeddings encourages the vision encoder to associate visual patterns in these regions with corresponding descriptors, reducing reliance on global shortcuts such as overall lung brightness or scanner-dependent artifacts, a phenomenon also reported in knowledge-enhanced pretraining frameworks. [35] This interpretation is consistent with the observed shift from high-sensitivity, low-specificity behavior in the unimodal baseline to more balanced decision boundaries in multimodal variants, especially during external validation, echoing prior evidence that image–text pretraining improves cross-dataset robustness in medical imaging. [36] Multimodal training with text supervision also constrains the geometry of the image feature space. During joint optimization, images paired with similar textual descriptions are pulled closer in the shared latent space, while those with different semantics are pushed apart, analogous to contrastive objectives used in medical vision–language pretraining. [36]

We observed that multimodal learning with clinical text supervision yielded an image head that outperformed the unimodal baseline on the internal Shenzhen set and, more importantly, generalized better to external MC, TBX11K, and NIAID TB Portals data, as reflected by higher discrimination metrics. Within the multimodal family, using deterministic structured reports derived from clinical metadata and notes consistently led to stronger performance and more clinically coherent visual explanations than using raw notes, paralleling observations from knowledge-enhanced CXR vision–language models that explicitly structure report content into entities or knowledge graphs. [35] Multimodal model trained with structured text exhibited tighter within-class clusters and larger separation between normal and TB-positive cases than the unimodal model, suggesting that the image embeddings have been reorganized around disease semantics rather than dataset-specific low-level cues. Raw notes capture reporting variability and comorbidities, while structured text distills this information into consistent categorical cues; together, these signals can regularize the image encoder away from overfitting to specific, non-generalizable, or noisy visual features, a strategy conceptually aligned with recent reviews emphasizing semantic fusion of imaging and clinical data. [37]

### Advantages of Structured Reports over Raw Clinical Notes

The performance comparison between using raw notes and structured reports illustrates how the design of the textual channel shapes the learned visual representation. Both multimodal models see the same CXRs and labels, yet the multimodal-structured model variant consistently achieved higher performances across internal and external test sets (Tables 3 and 4), indicating that the gains are driven by supervision quality. Structured reports were generated via a deterministic, rule-based pipeline that converts image metadata and brief clinical notes into a single canonical sentence. This is similar in spirit to a recent work that extracts entity-level descriptions (e.g., anatomy, findings, modifiers) from radiology reports before aligning them with images. [35] The empirical observation that structured text yielded notable improvements on external test cohorts supports the view that a standardized report representation provides a cleaner and more transferable alignment signal than raw narrative, consistent with recent findings that entity- or knowledge-level supervision can improve both discrimination and grounding of CXR vision–language models. [35]

The benefits of structured supervision were also evident in representation and explainability analyses. In the UMAP embedding space, the structured-text model showed tighter within-class clusters between normal and TB-positive CXRs than both the unimodal and multimodal-raw models (Figs. 4, 5 and 6). While UMAP is a qualitative visualization tool, the consistent visual separation across internal and external datasets is aligned with the observed metric improvements and with prior reports that vision–language pretraining yields more semantically structured embedding spaces for radiology tasks. [36]

Grad-CAM heatmaps provided a complementary view of how multimodal learning impacted spatial attention. Because structured reports explicitly encoded laterality and lobar involvement, the model repeatedly saw the same tokens co-occurring with characteristic radiographic patterns in specific regions, similar to entity-grounded model supervision. [38] Compared with the unimodal and multimodal-raw models, the multimodal-structured model frequently concentrated Grad-CAM activation within or directly adjacent to TB lesions and reduced off-target attention (Figs. 7, 8 and 9). These observations are qualitative, but they are consistent with the hypothesis that multimodal-structured learning encouraged the image head to focus on clinically meaningful regions, echoing findings from other CXR vision–language models that report improved grounding and interpretability when using structured or knowledge-enhanced textual supervision. [38, 39]

### Model Generalizability

Multimodal learning with structured notes also improved generalization to external cohorts, where acquisition conditions, patient populations, and reporting styles differ from the internal training data. The unimodal model tended to over-predict TB on external sets, achieving high sensitivity but poor specificity, indicative of overfitting, a domain-shift problem widely documented in computer-aided TB diagnosis. [21] In contrast, both multimodal models, and especially the multimodal-structured variant, achieved more balanced sensitivity–specificity trade-offs and higher discrimination metrics on the external test cohorts (Tables 3 and 4). This pattern is consistent with semantic regularization: by aligning images to text that encodes cross-institutionally stable TB attributes, including presence, status, subtype, location, and pattern, the model is less free to rely on site-specific low-level cues and more likely to learn features that transfer across domains, as also suggested by a recent multimodal pretraining work in chest radiography. [40]

## Conclusion, Limitations, and Future Work

Our study builds on the observation that TB radiographic signs are often subtle, heterogeneous, and affected by acquisition and population shifts, and that clinical text can provide complementary semantic cues during training. Multimodal training with text supervision that preserves sensitivity while improving specificity on external cohorts can help prioritize CXRs for expert review, reducing false positives without missing abnormal cases, which is an important goal for AI‑supported TB screening in resource‑limited settings. Our results show that multimodal training substantially improves TB classification, especially when using deterministic, structured reports. Text supervision during training reshapes image representations toward clinically meaningful semantics, leading to stronger external generalization and more lesion‑focused saliency maps than unimodal baselines.

We note that the goal of this study is to evaluate the effect of privileged structured-report supervision on generalizable image-only inference, rather than to perform an exhaustive benchmark across vision backbones. We therefore selected VGG-11 as a controlled and extensively validated CXR baseline to isolate the contribution of the proposed multimodal alignment objectives. Since the training objective operates on the projected image embedding rather than backbone-specific internals, the framework is directly compatible with stronger CNN families (e.g., DenseNet/ResNet-style variants) and vision transformers (e.g., ViT/Swin), and these can be substituted as drop-in image encoders under identical data splits and optimization protocol.

This study has several limitations that should be acknowledged. First, from the model standpoint, the study focuses on a particular choice of vision and text encoder, fixed image resolution, and fixed shared embedding dimensionality; other architectures, hyperparameters, and/or ensemble strategies might further improve robustness. Second, the study leverages only four TB CXR datasets that, while diverse, do not cover the full range of global TB prevalence, comorbidities, and imaging hardware. Therefore, broader validation is needed on additional cohorts, including private hospital and mobile screening populations. Third, our evaluation and discussion are limited to discrimination metrics, while model calibration and/or decision-curve analyses are excluded since these tend to be deployment-specific. Local validation and calibration to site‑specific prevalence and workflows are a necessary step for the practical use of this approach.

Thus, future work should extend the present analysis along several axes. Evaluating stronger vision encoders (e.g., DenseNet/ResNet variants and vision transformers such as ViT/Swin) and ensemble strategies under the same privileged structured-report supervision objective would help quantify how consistently the proposed alignment framework transfers across backbone families while preserving the deployment constraint of image-only inference. Established best practices, such as systematic calibration and model assessment (e.g., reliability diagrams, Brier scores, and calibration methods) could be used to determine how the alignment affects probability outputs and decision thresholds. Radiologist-in-the-loop studies are critical to assess clinical utility. Prospective reader experiments could determine whether text supervision during multimodal training improves diagnostic accuracy, reduces interpretation time, or enhances end-user confidence, and whether Grad-CAM explanations are concordant with expert reasoning. Controlled synthetic text augmentation using large language models, constrained by structured templates and subject to clinical review, could be explored to expand the textual supervision while minimizing hallucination risk. Finally, extending the approach beyond binary TB screening to multi-label thoracic disease classification and to other medical imaging modalities such as CT or ultrasound could test the generality of alignment-regularized unimodal inference in broader clinical contexts, in line with current efforts to build multimodal foundation models for medical imaging.

## Supplementary Information

Below is the link to the electronic supplementary material.

Supplementary Material 1 (DOCX 950 KB)

## Data Availability

All data used in this work are publicly available, and sources are cited in the article. The code is available at https://github.com/antani-lab/Multimodal-Structured-Report-Supervision-for-Unimodal-Inference-SRK.
